# Association of functional genetic variants in *TFF1* and nephrolithiasis risk in a Chinese population

**DOI:** 10.1186/s12894-022-01081-w

**Published:** 2022-08-20

**Authors:** Qiangdong Wang, Yan Jiang, Mulong Du, Lei Yang, Qinbo Yuan

**Affiliations:** 1 Department of Urology, Huaiyin Hospital of Huai’an City, Huai’an, China; 2 Jiangyin Center for Disease Control and Prevention, Jiangyin, China; 3 Department of Biostatistics, Center for Global Health, School of Public Health, Nanjing Medical University, Nanjing, China; 4 Department of Geriatrics, The Second People’s Hospital of Wuxi, 68 Zhongshan Road, Wuxi, China; 5 Department of Urology, The Fifth People’s Hospital of Wuxi, 1215 Guangrui Road, Wuxi, China

**Keywords:** *TFF1*, Nephrolithiasis, Single nucleotide polymorphism, Risk factors

## Abstract

Trefoil Factor 1 (*TFF1*) is considered to be able to inhibit the formation of kidney stone. However, genetic variants in *TFF1* and corresponding function in kidney stone development are still not well studied. In this study, the discovery set including 230 cases and 250 controls was used to analyze the association between seven tagSNPs of *TFF1* gene and the nephrolithiasis risk. Further evaluation was confirmed by the validation set comprising 307 cases and 461 controls. The consequences of the two-stage case–control study indicated that individuals with the rs3761376 A allele have significantly increased nephrolithiasis risk than those with the GG genotypes [adjusted odds ratio (OR) = 1.35, 95% confidence interval (CI) = 1.05–1.73]. Moreover, we also carried out a stratified analysis and found the increased nephrolithiasis risks at A allele among males, overweight individuals, no hypertensive individuals, nondiabetic individuals, smokers, and drinkers. In the following functional experiments, the notably lower expression of *TFF1* was exhibited by the vectors carrying A allele compared with those carrying G allele in both luciferase (*P* = 0.022) and expression vectors (*P* = 0.041). In addition to tissue detection, we confirmed a significant inverse association of rs3761376 G > A and *TFF1* gene expression (*P* < 0.001). These results suggest that *TFF1* rs3761376 may serve as a potential biomarker to predict the risk of nephrolithiasis.

**Supplementary Information:**

The online version contains supplementary material available at 10.1186/s12894-022-01081-w.

## Introduction

Kidney stone disease (also named nephrolithiasis) remains a public health problem around the world, of which the lifetime prevalence is approximately 10% in men and 5% in women [1, 2]. Of all types of renal stones, calcium oxalate (CaOx) is the most common composition found by chemical analysis [3]. Previous studies have revealed many factors associated with an increased risk of kidney stone formation, such as diabetes mellitus [4], metabolic syndrome [5], hypertension, and obesity [6]. In addition, genetic variation also played a role in the pathogenesis of kidney stone in the northeastern (NE) Thai population and the Russian population [7, 8], while few studies were performed in Chinese population.

So far, exact pathogenesis of kidney stone formation is still poorly understood. A long-standing hypothesis suggested that stones originally formed in the lumen of the renal tubules [9]. The calcium ions and oxalate ions in the urine of stone formers are usually supersaturated [10], and are conducive to the nucleation, growth and aggregation of CaOx crystals [11]. During this formation process, there are urine substances called "stone inhibitors" in the normal renal tubule fluid, which inhibit the formation of kidney stones [12]. These substances include proteins, lipids, glycosaminoglycans and inorganic compounds. Therefore, decreased levels and functional changes of these molecules (especially proteins) in renal tubule fluid and urine can thus lead to the development of kidney stones [13–15].

The mammalian trefoil factor family (TFF) peptides consist of a three-looped structure of cysteine residues, called trefoil domains, and the family includes three members in mammals: *TFF1*, *TFF2*, and *TFF3* [16, 17], playing an important role in the regeneration and repair of the urinary tract. *TFF1* can dimerize into a homodimer through a seventh cysteine residue [18]. These small peptides with a molecular weight of approximately 7 kDa are secreted by epithelial cells of various tissues, including renal tubular epithelial cells [19]. TFF peptides promote the process of epithelial recovery and regeneration by inducing cell migration, angiogenesis, and increasing cell resistance to pro-apoptotic stimuli [20–22]. Although TFF peptides have been mainly studied in the gastrointestinal tract, they were also detected in the urinary tract [23]. In addition, new research showed that *TFF1* could effectively inhibit the growth and aggregation of calcium oxalate monohydrate crystals [24]. A super-physiological concentration of 4 µg/ml *TFF1* could convert calcium oxalate monohydrate crystals into dihydrate type, and its adsorption capacity was much lower [25]. Besides, another study indicated that *TFF1* is a new and potent CaOx crystal growth inhibitor with potential pathophysiological effects in kidney stones, consistent with previous experiments [26]. However, the mechanism of regulating *TFF1* expression and its role in kidney stones still needs further investigations.

Single nucleotide polymorphisms (SNPs), a common source of genetic variation, may play a critical role in the occurrence of nephrolithiasis. A genome-wide association study (GWAS) by Thorleifsson et al. [27] that mapped the entire human genome concluded that among patients in Iceland and the Netherlands, claudin 14 (*CLDN14*) SNPs rs219780 and rs219781 of exon 7 are related to kidney stones. Guha et al. [28] found that calcium sensing receptor (*CaSR*) and CLDN14 gene polymorphisms are associated with increased risk of kidney stones in patients from the eastern part of India. In the Chinese population, multiple genes polymorphisms have been found to be significantly associated with the occurrence of kidney stones, such as G protein signaling 14 (*RGS14*) rs12654812, osteopontin (*OPN*) rs11439060 and migration inhibitory factor (*MIF*) rs755622 [29–31]. However, to date, there is no studies to evaluate the association between the *TFF1* polymorphisms and nephrolithiasis risk.

In this study, we selected seven tagSNPs of *TFF1* and assessed their association with nephrolithiasis risk by using a two-stage case–control study, followed by molecular biological experiments.

## Material and methods

### Study population

The study included two independent sets, which respectively is discovery set and validation set. There were 230 nephrolithiasis cases and 250 controls in the discovery set and the validation set contained 307 cases and 461 controls. All cases were confirmed to have nephrolithiasis by X-ray, B-ultrasonic and CT detection when enrolled from Huaiyin Hospital between March 2010 and January 2013. Patients who had neoplasm or with incomplete clinical data were excluded from the study. The controls were recruited from those seeking general physical examinations at the outpatient of the same hospital in the meanwhile and confirmed to be kidney stone free by medical history and urinary ultrasonography. Controls with a history of stone episodes and any evidence of urinary tract calculi were excluded from the study. The definition of smokers was those who smoked daily for more than one year. The status of body mass index (BMI), hypertension and diabetes are on the basis of the World Health Organization standards. We obtained the information of individual demographics through face-to-face interviews. Demographic details could be found in Additional file 1: Table S1 and Additional file 2: S2.

### SNP selection and genotyping

We followed previous study by Wang et al. [32] to select seven tagSNPs of *TFF1* (i.e., rs225355, rs2839488, rs13051704, rs225358, rs3761376, rs225359, rs35448902). Briefly, clear linkage disequilibrium was observed in these variants (*r*^2^ > 0.8) by Haploview 4.0 and the minor allele frequency (MAF) of the 7 selected SNPs were over 0.05 in the population of CHB (Han Chinese in Beijing, China [CHB]) from the 1000 Genomes Project. In both discovery set and validation set, genomic DNA was extracted from blood cell. By using ABI 7900HT real-time polymerase chain reaction (PCR) system (Applied Biosystems, Foster City, CA), TaqMan SNP Genotyping Assay was carried out to detect the genotypes of the tagSNPs. Randomly selecting 10% of the samples from both sets for being re-genotyped, we found that the concordance rate was 100.0%.

### Cell lines and cell culture

In this study, we used a nephrolithiasis cell line (HEK-293), which was purchased from Shanghai Institute of Biochemistry and Cell Biology, Chinese Academy of Sciences (Shanghai, China). All these cells were cultured in Roswell Park Memorial Institute 1640 medium by adding 100 U/ml penicillin, 10% fetal bovine serum and 100 µg/ml streptomycin (Life Technologies/Gibco, Grand Island, NY) at 37 °C in a humidified atmosphere with 5% CO_2_.

### Luciferase assay by construction of plasmids and transfection

We carried out two kinds of luciferase experiment by respectively synthesizing and constructing two different sequences into pGL3-basic vector (Promega, Madison, WI) in luciferase reporter plasmids. One sequence contained the 1000-bp upstream fragment of the transcription start site of *TFF1* with different alleles of rs3761376, and the other sequence involved the intact gene fragment of *TFF1* with different alleles of rs3761376. By cloning both the synthesized *TFF1* coding sequences into pcDNA3.1 expression vector (Promega, Madison, WI), the *TFF1* over-expression vector was constructed. All the plasmids were provided by Generay Company (Shanghai, China), and were verified by DNA sequencing. Then, to detect the luciferase activity, we transfected the luciferase reporter plasmids into cells by Lipofectamine 2000 (Invtrogen, Carlsbad, CA). After 24 h, the luciferase activity was measured on the Dual-Luciferase Reporter Assay System (Promega, Madison, WI) and we calculate the expression by the ratio of firefly luciferase to Renilla luciferase activities.

### Real-time PCR

Normal kidney tissues were obtained from 52 suspected nephritis patients (actually without nephritis) who had undergone kidney puncture biopsy. RNAlater protection solution was added to store the kidney tissues on ice, and total RNA was extracted from cells and kidney tissues by Trizol Reagent (Invitrogen) later. After reverse transcription, we detected the expression of mRNA by real-time PCR (ABI 7300) with SYBR Green assay (TaKaRa Biotechnology, Dalian, China). Each reaction was performed in triplicate, and the primers are provided as listed in Additional file 3: Table S3. The corresponding genotyping of 52 cases was performed via TaqMan SNP Genotyping Assay as above.

### Statistical analysis

The Pearson's χ^2^ test and Student's *t*-test were used to evaluate the distribution differences of the demographic characteristics between cases and controls. We estimated the association between *TFF1* tagSNPs and nephrolithiasis risk by using adjusted odds ratios (ORs) and 95% confidence intervals (95% CIs) from logistic regression analysis. Subgroup analysis was conducted by age (≤ 46 or > 46 years), gender (male or female), BMI (≤ 24 or > 24 kg/m^2^), hypertension (yes or no), diabetes (yes or no), smoking status (ever or never) and drinking status (ever or never). Heterogeneity among these factors were calculated and we thought there was no significant heterogeneity when *P*_heterogeneity_ > 0.10. The interaction analysis of tagSNPs and these factors was also completed by the logistic regression. Differences in luciferase activity and *TFF1* expression level were assessed by Student's t-test. All the statistical analyses were constructed with the SPSS software version 22.0 and Stata software version 16, and *P* < 0.05 for two-side analysis was identified as a statistically significant difference.

### Sample size analysis

Using the sample size calculation website (http://powerandsamplesize.com/Calculators/Compare-2-Proportions/2-Sample-Equality) provided by HyLown Consulting, based on the MAF = 0.471, 0.366, the odds ratio (OR) = 1.35, α = 5%, and power (1 − β) = 80%, the sample size stated a number of at least 296 for each group.

## Results

### Characteristics of the study population

Table 1 represented the information of demographic characteristics of the combined set, which included 537 cases and 711 controls. There was no significant difference between cases and controls among gender, BMI, diabetes, and drinking status (*P* = 0.656, 0.113, 0.934 and 0.996). Yet, on average, the cases were older than the controls (*P* = 0.007) and the cases included more hypertensive individuals and smokers than controls (*P* < 0.001).

**Table 1:** The distribution of the demographic characteristics of combined set

Variables	Cases, n = 537	Controls, n = 711	*P*-value^a^
Mean age ± SD, years	48.0 ± 13.2	46.1 ± 9.4	**0.007**
≤ 46, n (%)	243 (45.3)	243 (53.0)	
> 46, n (%)	294 (54.7)	218 (47.0)	
Gender, n (%)			0.656
Male	363 (67.7)	473 (66.5)	
Female	173 (32.3)	238 (33.5)	
Body mass index, n (%)			0.113
≤ 24	231 (45.5)	356 (50.1)	
> 24	277 (54.5)	355 (49.9)	
Hypertension, n (%)			**< 0.001**
Yes	144 (27.8)	130 (18.3)	
No	374 (72.2)	580 (81.7)	
Diabetes, n (%)			0.934
Yes	30 (5.8)	42 (5.9)	
No	487 (94.2)	668 (94.1)	
Smoking status, n (%)			**< 0.001**
Ever	221 (41.5)	215 (30.3)	
Never	312 (58.5)	495 (69.7)	
Drinking status, n (%)			0.996
Ever	188 (35.3)	251 (35.4)	
Never	344 (64.7)	459 (64.6)	

Bold font indicates *P* values < 0.05, which were statistically significant

^a^*P*-value for two-sided χ^2^ test. SD, standard deviation

### Association between *TFF1* tagSNPs and nephrolithiasis risk

Genotype frequencies of *TFF1* tagSNPs of the cases and controls and their risk contributing to nephrolithiasis were listed in Table 2. In the discovery set, the association between rs3761376 and the increased risk of nephrolithiasis was found in the analyses of additive, dominant and recessive genetic model, and this association existed after performing Bonferroni correction. In addition, for rs13051704 and rs35448902, a reduced incidence of nephrolithiasis appeared in additive and dominant genetic model, while these significant differences disappeared after performing Bonferroni correction. To confirm the association, we conducted an analysis in validation set. The results showed that rs3761376 was still associated with nephrolithiasis risk in additive and recessive genetic model, which was in line with the results of validation set. In detail, individuals with the rs3761376 AA genotype confronted greater nephrolithiasis risk compared with those with GG genotype in co-dominant genetic model (*P* = 0.007, adjusted OR = 1.83, 95% CI = 1.18–2.84). Carriers of rs3761376 G allele genotypes [GG and AG] were under-represented in the nephrolithiasis patients compared with the controls. Hence, individuals with the rs3761376 G allele genotypes [GG and AG] had a significantly lower nephrolithiasis risk compared to those with the AA genotype (*P* = 0.002, adjusted OR = 1.86, 95% CI = 1.25–2.78). Finally, the results of the combined set also explicitly manifested the increased nephrolithiasis risk effected by A allele of rs3761376 in additive, dominant and recessive genetic model.

**Table 2:** Association of *TFF1* and nephrolithiasis risk in each stage

Stage	tagSNPs	Alleles (major/minor)	Cases^a^	Controls^a^	Adjusted OR (95% CI)^b^							
					Co-dominant model		Additive model	Dominant model	Recessive model	*P*^c^	*P*_adj_^d^	*P*_trend_
					Het^b^	Hom^b^						
Discovery	rs225355	G > A	147/72/11	151/93/6	0.74 (0.50–1.09)	2.00 (0.70–5.75)	0.81 (0.55–1.18)	0.81 (0.55–1.18)	2.23 (0.78–6.34)	0.268	1.000	0.185
	rs2839488	C > G	129/79/19	147/86/16	1.01 (0.68–1.51)	1.50 (0.72–3.10)	1.12 (0.84–1.51)	1.08 (0.74–1.57)	1.49 (0.73–3.04)	0.433	1.000	0.594
	rs13051704	C > G	140/63/10	131/107/12	**0.53** **(0.35–0.79)**	0.70 (0.29–1.73)	**0.64** **(0.46–0.89)**	**0.54** **(0.37–0.80)**	0.90 (0.37–2.17)	**0.008**	0.056	**< 0.001**
	rs225358	C > T	128/82/20	143/79/28	1.21 (0.81–1.81)	0.82 (0.43–1.56)	1.01 (0.76–1.32)	1.11 (0.77–1.61)	0.77 (0.41–1.43)	0.969	1.000	0.499
	rs3761376	G > A	64/112/51	100/117/33	1.47 (0.97–2.23)	**2.21** **(1.28–3.85)**	**1.48** **(1.14–1.94)**	**1.64** **(1.10–2.43)**	**1.76** **(1.08–2.88)**	**0.004**	**0.028**	**0.003**
	rs225359	G > A	121/90/19	144/84/22	1.36 (0.92–2.02)	1.05 (0.53–2.07)	1.15 (0.86–1.53)	1.30 (0.90–1.88)	0.93 (0.48–1.80)	0.335	1.000	0.451
	rs35448902	C > T	151/65/11	140/97/13	**0.63** **(0.43–0.94)**	0.72 (0.30–1.69)	**0.72** **(0.53–0.99)**	**0.64** **(0.44–0.94)**	0.84 (0.36–1.96)	**0.043**	0.301	**0.028**
Validation	rs3761376	G > A	108/123/66	186/214/61	0.97 (0.69–1.36)	**1.83** **(1.18–2.84)**	**1.28** **(1.03–1.58)**	1.16 (0.85–1.59)	**1.86** **(1.25–2.78)**	**0.026**	**–**	**< 0.001**
Combined	rs3761376	G > A	172/235/117	286/331/94	1.15 (0.88–1.51)	**2.01** **(1.42–2.85)**	**1.37** **(1.16–1.62)**	**1.35** **(1.05–1.73)**	**1.86** **(1.35–2.54)**	**< 0.001**	**–**	**< 0.001**

Bold font indicates *P* values < 0.05, which were statistically significant

^a^Major homozygote/heterozygote/rare homozygote between cases and controls

^b^Logistic regression model with adjustment for age, sex, hypertension and smoking status in co-dominant (het: heterozygote vs. major homozygote; hom: rare homozygote vs. major homozygote), additive (rare homozygote vs. heterozygote vs. major homozygote), dominant (heterozygote/rare homozygote vs. major homozygote), recessive (rare homozygote vs. heterozygote / major homozygote) models; OR, odds ratio; CI, confidence interval

^c^*P* for additive model

^d^*P*_adj_ adjusted by Bonferroni correction

### Stratification analysis of association between the rs3761376 and nephrolithiasis risk

We conducted the stratified analyses by age, gender, BMI, hypertension, diabetes, smoking and drinking status, basing on the dominant genetic model of the rs3761376 in the combined set. As showed in Table 3, increased nephrolithiasis risks for the A allele genotypes [AA and AG] were observed among males (adjusted OR = 1.50, 95% CI = 1.09–2.08, *P* = 0.013), overweight individuals (adjusted OR = 1.55, 95% CI = 1.09–2.22, *P* = 0.015), not hypertensive individuals (adjusted OR = 1.35, 95% CI = 1.01–1.79, *P* = 0.041), nondiabetic individuals (adjusted OR = 1.34, 95% CI = 1.04–1.74, *P* = 0.027), smokers (adjusted OR = 1.68, 95% CI = 1.09–2.59, *P* = 0.020) and drinkers (adjusted OR = 2.32, 95% CI = 1.48–3.64, *P* < 0.001). For the test of heterogeneity, most factors had low heterogeneity, instead the heterogeneity of drinking status was high (*P*_heterogeneity_ = 0.005). An obvious increase in nephrolithiasis risk existed with the interaction between polymorphisms of *TFF1* and drinking status (*P*_interaction_ = 0.037).

**Table 3:** Stratified analyses on the association between *TFF1* rs3761376 and nephrolithiasis risk

Characteristic	Cases (n = 537), n (%)		Controls (n = 711), n (%)		OR (95% CI)^a^	*P*^a^	*P*_heterogeneity_	*P*_interaction_
	GG	AG + AA	GG	AG + AA				
Age, years							0.744	0.152
≤ 46	73 (30.8)	164 (69.2)	151 (40.1)	226 (59.9)	1.44 (0.98–2.10)	0.062		
> 46	99 (34.5)	188 (65.5)	135 (40.4)	199 (59.6)	1.32 (0.92–1.88)	0.135		
Gender							0.806	0.562
Male	109 (30.6)	247 (69.4)	183 (38.7)	290 (61.3)	**1.50 (1.09–2.08)**	**0.013**		
Female	63 (37.5)	105 (62.5)	103 (43.3)	135 (56.7)	1.61 (0.73–1.85)	0.528		
BMI, mg/k^2^							0.392	0.345
≤ 24	78 (34.7)	147 (65.3)	144 (40.4)	212 (59.6)	1.24 (0.86–1.79)	0.241		
> 24	80 (29.3)	193 (70.3)	142 (40.0)	213 (60.0)	**1.55 (1.09–2.22)**	**0.015**		
HP							0.981	0.823
Yes	45 (31.5)	98 (68.5)	47 (36.2)	83 (63.8)	1.36 (0.80–2.30)	0.255		
No	122 (33.6)	241 (66.4)	238 (41.0)	342 (59.0)	**1.35 (1.01–1.79)**	**0.041**		
Diabetes							0.968	0.882
Yes	13 (43.3)	17 (56.7)	20 (47.6)	22 (52.4)	1.37 (0.47–3.98)	0.562		
No	152 (32.0)	323 (68.0)	265 (39.7)	403 (60.3)	**1.34 (1.04–1.74)**	**0.027**		
Smoking status							0.237	0.484
Ever	64 (29.6)	152 (70.4)	82 (38.1)	133 (61.9)	**1.68 (1.09–2.59)**	**0.020**		
Never	107 (35.2)	197 (64.8)	203 (41.0)	292 (59.0)	1.22 (0.90–1.66)	0.203		
Drinking status							**0.005**	**0.037**
Ever	49 (26.8)	134 (73.2)	105 (41.8)	146 (58.2)	**2.32 (1.48–3.64)**	**< 0.001**		
Never	122 (36.3)	214 (63.7)	180 (39.2)	279 (60.8)	1.06 (0.78–1.44)	0.733		

Bold font indicates *P* values < 0.05, which were statistically significant

^a^Adjusted for age, sex, hypertension and smoking status as appropriate in the logistic regression model; BMI, body mass index; HP, hypertension

### Effects of rs3761376 on transcriptional activity

**Fig. 1:**
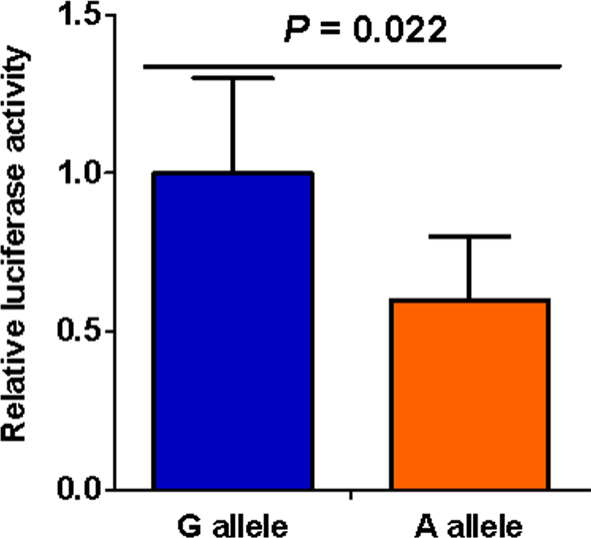
Luciferase activity affected by rs3761376. Different luciferase reporter vectors with either rs3761376 allele (G or A) which included the sequence of the promoter of *TFF1* were transfected into HEK-293 cells. Then, the luciferase activity was detected and normalized by the internal control of Renilla luciferase

**Fig. 2:**
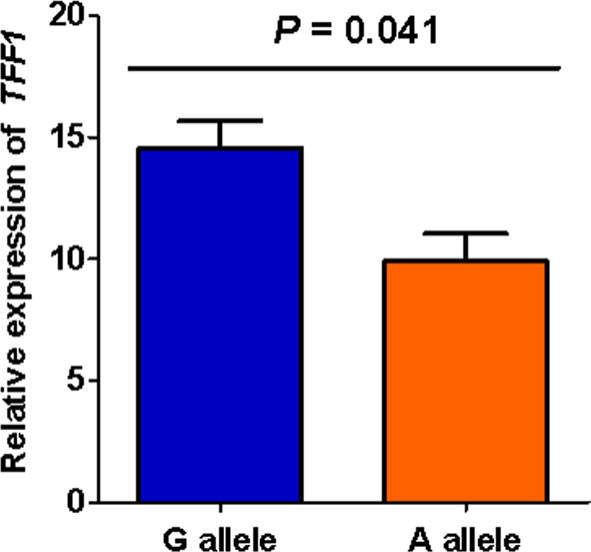
Transcriptional activity affected by rs3761376. Different expression vectors with either rs3761376 allele (G or A) which included the sequence of the promoter of *TFF1* were transfected into HEK-293 cells. Then, the transcriptional activity was detected by real-time PCR

To evaluate the genetic effect on *TFF1* gene expression, we first constructed the sequence including the promoter of *TFF1* with either rs3761376 allele (G or A) into different luciferase reporter vectors, and then transfected the vectors into HEK-293 cells. As presented in Fig. 1, the vectors carrying A allele had a significantly reduced luciferase activities compared to those carrying G allele (*P* = 0.022). Next, the synthesized fragment of *TFF1* with either G or A rs3761376 allele was inserted into expression plasmid. In Fig. 2, the results showed the lower transcriptional activities was exhibited by the vectors carrying A allele than by those carrying G allele (*P* = 0.041).

### Correlation of rs3761376 genotypes with expression levels of *TFF1*

**Fig. 3:**
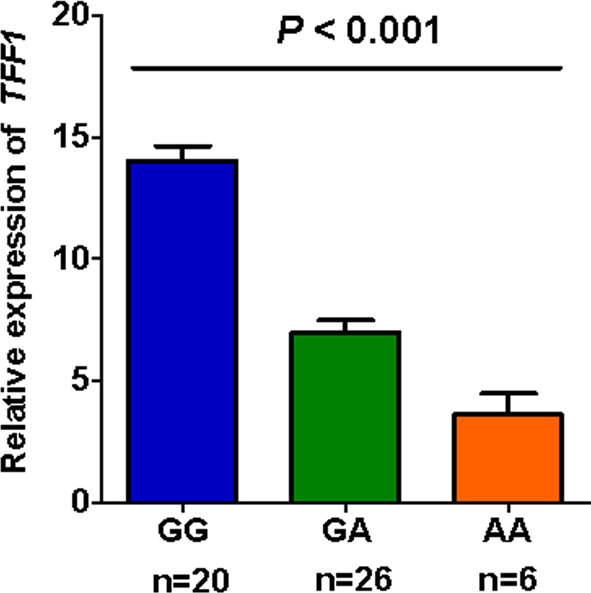
Correlation between different rs3761376 genotypes and *TFF1* expression

For further study of the rs3761376 effects, we evaluated the expression levels of *TFF1* with known genotypes in 52 kidney tissues of nephrolithiasis patients. In Fig. 3, the rs3761376 A allele significantly lower *TFF1* expression (*P* < 0.001), which was in accordance with the results of the cell experiments.

## Discussion

As a novel potent CaOx crystal growth inhibitor, *TFF1* may play a potential pathophysiological role in nephrolithiasis [25, 26]. Numerous evidences have showed that genetic variant had a major effect for the incidence of cancer [33–35]. To date, few studies were taken to prove the association between the *TFF1* polymorphisms and nephrolithiasis risk. Hence, we performed a two-stage case–control study to evaluate this association and found that individuals with the rs3761376 AA genotype confronted significantly greater nephrolithiasis risk. In stratified analyses, increased nephrolithiasis risks for the A allele genotypes [AA and AG] were observed among males, overweight individuals, not hypertensive individuals, nondiabetic individuals, smokers and drinkers.

In China, it is a fact that the proportion of drinking and smoking is higher in male than in female; in addition, the prevalence of kidney stones in male is higher than in female, which may lead to different risks among the gender [36]. Other results of the stratified analysis indicate that the influence of genetic variants in *TFF1* on the risk of nephrolithiasis may be regulated by specific demographic factors and environmental exposures. A possible explanation is that some environmental hazards, such as smoking and drinking, increase the risk of kidney stones, thereby enhancing the effect of rs3761376 variants. This also provides evidence that the pathogenesis is a complex process involving both genetic and environmental factors [37].

For further genetic function, we carried out two vector assays and discovered that the vectors carrying rs3761376 A allele both had a significantly reduced luciferase and transcriptional activities compared to those carrying G allele. Rs3761376 was located in the promoter of *TFF1* and polymorphisms in promoter region were reported possessing potential functions to affect the transcription activity by altering the bindings of transcription factors [38–40]. Therefore, the results suggested that the A allele might reduce the promoter activity of *TFF1* by changing the bindings of transcription factors. Moreover, by evaluating the expression levels of *TFF1* with known genotypes in 52 kidney tissues of nephrolithiasis patients, a significantly lower *TFF1* expression was found along with the increase of the rs3761376 A allele. Combined the results of cell and tissue experiments, it is plausible that *TFF1* rs3761376 may have the ability to alter the bindings of transcription factors and modulate the *TFF1* expression. Studies have shown that the C-terminus of *TFF1* binds to Ca^2+^, which facilitates the entrapment of Ca^2+^ ions, resulting in the CaOx crystal growth inhibition [26, 41]. It suggests that the low expression of *TFF1* at the kidney may lead to the increase of CaOx crystals, contributing to the increased risk of nephrolithiasis.

Nonetheless, considered the complex process for kidney stone formation, it is impossible that any single SNP or gene would exert a clear influence on nephrolithiasis risk. Although a strong correlation between rs3761376 and nephrolithiasis risk was observed, we didn’t explore the potential effect of rs3761376 on regulation of *TFF1*. Many previous researches indicated that *TFF1* was an estrogen-induced protein and rs3761376 was capable of changing the motifs of specified transcription factors, especially estrogen receptor 1 (*ESR1*) [32, 42, 43]. Therefore, to verify the association between the *TFF1* genetics and nephrolithiasis risk in terms of mechanism, we need do more well-designed functional study. Moreover, some studies have pointed out that a multitude of comorbidities (hyperlipidemia, cardiovascular disease, chronic kidney disease) are associated with an increased risk of kidney stone disease [44–46], but we did not include patients’ complications, which limited the stratified analysis for the genetic effect. To explore potential pathogenic mediators, Tanikawa et al. [47] reported association of 10 urolithiasis loci with metabolic traits (BMI, total cholesterol, triglyceride, and blood sugar), kidney-related traits (blood urea nitrogen, serum creatinine, estimated glomerular filtration rate, and uric acid), and electrolytes (potassium [K], chloride [Cl], calcium [Ca], and phosphate [P]). However, the various relevant clinical biochemical characteristics were not collected in our study, and it is unclear whether the association of *TFF1* tagSNPs with nephrolithiasis is independent of these factors. In addition, we could not detect the *TFF1* levels in the kidney samples of the controls, so it is impossible to evaluate the difference in the expression levels of *TFF1* between the patients and the controls, and it’s difficult to explore the effects of tagSNPs specifically through *TFF1* causing kidney stones.

In summary, our study provided evidence that the *TFF1* promoter activity reduced by rs3761376 A allele could at least partially lead to the decreased *TFF1* expression and ultimately increase the risk of kidney stones. The results show that SNP rs3761376 can be used as a potential biomarker for predicting the risk of kidney stones in Chinese populations.

## Supplementary Information

**Additional file 1: Table S1**. The distribution of the demographic characteristics of discovery set.

**Additional file 2: Table S2**. The distribution of the demographic characteristics of validation set.

**Additional file 3: Table S3**. The primers applied for reverse transcription

## Data Availability

The datasets generated and/or analysed during the current study, that included genotyping and gene expression, are available in the GitHub repository, https://github.com/omics-mining-group/tff1_nephrolithiasis. All supporting data are available from the corresponding author upon reasonable request.
