# Supplementary material for: Association of functional genetic variants in TFF1 and nephrolithiasis risk in a Chinese population
Source: BMC Urol. 2022 Aug 20;22:127. doi: 10.1186/s12894-022-01081-w (PMC9392923; doi:10.1186/s12894-022-01081-w)
Supplement: Supplementary file 1 — Additional file 1: Table S1. The distribution of the demographic characteristics of discovery set. [file 12894_2022_1081_MOESM1_ESM.docx]

**Table S1**. The distribution of the demographic characteristics of discovery set.

| **Variables** | **Cases,**  **n=230** | **Controls,**  **n=250** | ***P*-value^1^** |
| --- | --- | --- | --- |
| Mean age ± SD,  years | 46.7 ± 12.5 | 45.9 ± 12.5 | 0.174 |
| ≤46, n (%) | 109 (47.4) | 134 (53.6) |  |
| ＞46, n (%) | 121 (52.6) | 116 (46.4) |  |
| Gender, n (%) |  |  | 0.785 |
| Male | 150 (65.2) | 166 (66.4) |  |
| Female | 80 (34.8) | 84 (33.6) |  |
| Body mass index, n (%) |  |  | 0.578 |
| ≤24 | 111 (48.3) | 127 (50.8) |  |
| ＞24 | 119 (51.7) | 123 (49.2) |  |
| Hypertension,  n (%) |  |  | 0.055 |
| Yes | 59 (25.7) | 46 (18.4) |  |
| No | 171 (74.3) | 204 (81.6) |  |
| Diabetes, n (%) |  |  | 0.931 |
| Yes | 17 (7.4) | 19 (7.6) |  |
| No | 213 (92.6) | 231 (92.4) |  |
| Smoking status,  n (%) |  |  | **0.001** |
| Ever | 103 (44.8) | 76 (30.4) |  |
| Never | 127 (55.2) | 174 (69.6) |  |
| Drinking status,  n (%) |  |  | 0.811 |
| Ever | 88 (38.3) | 93 (37.2) |  |
| Never | 142 (61.7) | 157 (62.8) |  |
| ${}^{1}{}$*P*-value for two-sided χ^2^ test. SD, standard deviation. | | | |
