# Supplementary material for: Association of functional genetic variants in TFF1 and nephrolithiasis risk in a Chinese population
Source: BMC Urol. 2022 Aug 20;22:127. doi: 10.1186/s12894-022-01081-w (PMC9392923; doi:10.1186/s12894-022-01081-w)
Supplement: Supplementary file 2 — Additional file 2: Table S2. The distribution of the demographic characteristics of validation set. [file 12894_2022_1081_MOESM2_ESM.docx]

**Table S2**. The distribution of the demographic characteristics of validation set.

| **Variables** | **Cases,**  **n=307** | **Controls,**  **n=461** | ***P*-value^1^** |
| --- | --- | --- | --- |
| Mean age ± SD,  years | 49.0 ± 13.5 | 46.2 ± 7.2 | **0.014** |
| ≤46, n (%) | 134 (43.6) | 243 (52.7) |  |
| ＞46, n (%) | 173 (56.4) | 218 (47.3) |  |
| Gender, n (%) |  |  | 0.382 |
| Male | 213 (69.6) | 307 (66.6) |  |
| Female | 93 (30.4) | 154 (33.4) |  |
| Body mass index, n (%) |  |  | 0.096 |
| ≤24 | 121 (43.4) | 229 (49.7) |  |
| ＞24 | 158 (56.6) | 232 (50.3) |  |
| Hypertension,  n (%) |  |  | **＜0.001** |
| Yes | 85 (29.5) | 84 (18.3) |  |
| No | 203 (70.5) | 376 (81.7) |  |
| Diabetes, n (%) |  |  | 0.770 |
| Yes | 13 (4.5) | 23 (5.0) |  |
| No | 274 (95.5) | 437 (95.0) |  |
| Smoking status,  n (%) |  |  | **0.013** |
| Ever | 118 (38.9) | 139 (30.2) |  |
| Never | 185 (61.1) | 321 (69.8) |  |
| Drinking status,  n (%) |  |  | 0.725 |
| Ever | 100 (33.1) | 158 (34.3) |  |
| Never | 202 (66.9) | 302 (65.7) |  |
| ${}^{1}{}$*P*-value for two-sided χ^2^ test. SD, standard deviation. | | | |
