# Supplementary material for: Association of functional genetic variants in TFF1 and nephrolithiasis risk in a Chinese population
Source: BMC Urol. 2022 Aug 20;22:127. doi: 10.1186/s12894-022-01081-w (PMC9392923; doi:10.1186/s12894-022-01081-w)
Supplement: Supplementary file 3 — Additional file 3: Table S3. The primers applied for reverse transcription [file 12894_2022_1081_MOESM3_ESM.docx]

**Table S3**. The primers applied for reverse transcription

|  | Sense (5'-3') | Anti-sense (5'-3') |
| --- | --- | --- |
| *TFF1* | GGTCCTGGTGTCCATGCTG | ACAGCAGCCCTTATTTGCAC |
| *β-actin* | GCATCGTCACCAACTGGGC | ACCTGGCCGTCAGGCAGCTC |
